# Evaluation of exposure-specific risks from two independent samples: A simulation study

**DOI:** 10.1186/1471-2288-11-1

**Published:** 2011-01-05

**Authors:** William M Reichmann, David Gagnon, C Robert Horsburgh, Elena Losina

**Affiliations:** 1 Department of Orthopedic Surgery, Brigham and Women's Hospital, 75 Francis Street, Boston, MA 02115, USA; 2 Department of Biostatistics, Boston University School of Public Health, 801 Massachusetts Avenue, Boston, MA 02118, USA; 3 Department of Epidemiology, Boston University School of Public Health, 715 Albany Street, Boston, MA 02118, USA; 4 Massachusetts Veterans Epidemiology Research and Information Center, VA Cooperative Studies Program, Veterans Affairs Medical Center, 150 S. Huntington Ave, Jamaica Plain, MA 02130, USA

## Abstract

**Background:**

Previous studies have proposed a simple product-based estimator for calculating exposure-specific risks (ESR), but the methodology has not been rigorously evaluated. The goal of our study was to evaluate the existing methodology for calculating the ESR, propose an improved point estimator, and propose variance estimates that will allow the calculation of confidence intervals (CIs).

**Methods:**

We conducted a simulation study to test the performance of two estimators and their associated confidence intervals: 1) current (simple product-based estimator) and 2) proposed revision (revised product-based estimator). The first method for ESR estimation was based on multiplying a relative risk (RR) of disease given a certain exposure by an overall risk of disease. The second method, which is proposed in this paper, was based on estimates of the risk of disease in the unexposed. We then multiply the updated risk by the RR to get the revised product-based estimator. A log-based variance was calculated for both estimators. Also, a binomial-based variance was calculated for the revised product-based estimator. 95% CIs were calculated based on these variance estimates. Accuracy of point estimators was evaluated by comparing observed relative bias (percent deviation from the true estimate). Interval estimators were evaluated by coverage probabilities and expected length of the 95% CI, given coverage. We evaluated these estimators across a wide range of exposure probabilities, disease probabilities, relative risks, and sample sizes.

**Results:**

We observed more bias and lower coverage probability when using the existing methodology. The revised product-based point estimator exhibited little observed relative bias (max: 4.0%) compared to the simple product-based estimator (max: 93.9%). Because the simple product-based estimator was biased, 95% CIs around this estimate exhibited small coverage probabilities. The 95% CI around the revised product-based estimator from the log-based variance provided better coverage in most situations.

**Conclusion:**

The currently accepted simple product-based method was only a reasonable approach when the exposure probability is small (< 0.05) and the RR is ≤ 3.0. The revised product-based estimator provides much improved accuracy.

## Background

Exposure-specific risk (ESR) is defined as the risk of disease (or any outcome) given a specific exposure (or subgroup). ESRs are useful to clinicians because it allows a much more meaningful way of explaining risk to patients. They are also useful to investigators who are looking to use ESRs for their own work, which may include publishing their own work or planning studies. In the absence of having access to the primary data or a reported estimate of the ESR in the literature, the ESR can be estimated from two independent samples if the investigator knows the overall risk of disease and the relative risk (RR) of disease given the exposure of interest. There have been a number of published studies where ESRs have been calculated from two independent samples by multiplying the overall risk of disease from one sample by the RR from a second independent sample [1,2]. Stewart et al. computed the ESR of hip fracture given certain exposures (prior fracture, family history of fracture, low body weight, and smoking) in persons over the age of 70 in the United Kingdom [2]. This study found that the ESR of hip fracture among those with all 4 exposures was 8.9%. This was done by multiplying an overall risk of hip fracture of 1.91% by a RR of 4.66 [2].

Horsburgh computed the ESR of tuberculosis for multiple risk factors, along with 95% confidence intervals (CIs). The upper (lower) bound of the 95% CI for the ESR was calculated by multiplying the upper (lower) bound of the 95% CI for the overall risk by the upper (lower) bound of the 95% CI for the RR [1]. While there has been some work addressing the multiplication of two binomial parameters [3], to the best of our knowledge, there are no methodological articles evaluating the properties of the simple product-based estimator that was used in the articles by Stewart et al and Horsburgh.

In this article we set to address three objectives. The first is to evaluate the properties of the simple product-based estimator of the ESR used by Stewart et al and Horsburgh. The second objective is to propose an estimate of the variance of the ESR, which can subsequently be used for calculating 95% CIs. Lastly, we propose a revised product-based estimator and two variances estimates for the revised point estimator which are used to calculate 95% CIs.

## Methods

### Overview

We designed and implemented a simulation study to examine the properties of two different estimators of the ESR and their 95% CIs. The two estimators we sought to evaluate (and their associated CIs) were a simple product-based estimator and revised product-based estimator. Point estimators were evaluated by calculating the observed relative bias. Their 95% CIs were evaluated using coverage probabilities and expected length given coverage for a wide range of parameters, including exposure probability, probability of disease among the unexposed, the RR of disease given exposure, and the sample size.

For the purposes of this paper, D represents having disease, E represents having exposure, subscript one denotes that the quantity comes from sample one, and subscript two denotes that the quantity comes from sample two. More careful examination of the mathematics behind this simple product-based estimator clearly shows that the estimator is at best a crude approximation of the ESR.

(1) $${\text{P}}_{\text{1}}{\text{(D)*RR}}_{\text{2}}={\text{P}}_{\text{1}}\text{(D)*}\frac{{\text{P}}_{\text{2}}\text{(D|E)}}{{\text{P}}_{\text{2}}\text{(D|}\bar{\text{E}})}\neq \text{P(D|E)}=\text{ESR}$$

To estimate the ESR, this formula needs an estimate of the risk of disease in the unexposed (${\text{P}}_{\text{1}}\text{(D|}\bar{\text{E}})$) rather than the estimate of the overall risk of disease (P_1_(D)).

### Simple Product-Based ESR

The simple product-based ESR (denoted ESR_S_) is computed by simply multiplying the overall probability of disease from sample one by the RR from sample two. The formula is given below.

(2) $${\text{ESR}}_{\text{S}}={\text{P}}_{\text{1}}{\text{(D)*RR}}_{\text{2}}={\text{P}}_{\text{1}}\text{(D)*}\frac{{\text{P}}_{\text{2}}\text{(D|E)}}{{\text{P}}_{\text{2}}\text{(D|}\bar{\text{E}})}$$

### Variance and Confidence Interval for the Simple Product-Based ESR

We first propose a formula for the variance of the simple product-based estimator using a natural log transformation. We assumed that the covariance between ln(P_1_(D)) and ln(RR_2_) was zero because they are estimated from independent data sets.

(3) $$\begin{array}{l} {\text{Var(ln(ESR}}_{\text{S}}\text{))}={\text{Var(ln(P}}_{\text{1}}{\text{(D)*RR}}_{\text{2}}\text{))}\\ ={\text{Var(ln(P}}_{\text{1}}\text{(D))}+{\text{ln(RR}}_{\text{2}}\text{))}\\ ={\text{Var(ln(P}}_{\text{1}}\text{(D)}+{\text{Var(ln(RR}}_{\text{2}}\text{)) }\\ \;+{\text{2*Cov(ln(P}}_{\text{1}}{\text{(D)), ln(RR}}_{\text{2}}\text{))}\\ ={\text{Var(ln(P}}_{\text{1}}\text{(D)))}+{\text{Var(ln(RR}}_{\text{2}}\text{)) }\\ \text{ (assuming independence)}\\ ={\text{Var(ln(P}}_{\text{1}}\text{(D))) }+\frac{\text{1}-{\text{P}}_{\text{2}}\text{(D|E)}}{{\text{P}}_{\text{2}}{\text{(D|E)*n}}_{\text{2E}}}+\frac{\text{1}-{\text{P}}_{\text{2}}\text{(D|}\bar{\text{E}}\text{)}}{{\text{P}}_{\text{2}}\text{(D|}\bar{\text{E}}{\text{)*n}}_{\text{2}\bar{\text{E}}}} \end{array}$$

To complete the formula for the variance of the natural log ESR we need the variance of the natural log of the overall risk (Var(In(P_1_(D)))). This is derived below using the delta method.

(4) $$\begin{array}{c} {\text{Var(ln(P}}_{\text{1}}\text{(D)))}=\frac{\text{1}}{{\text{P}}_{\text{1}}{\text{(D)}}^{\text{2}}}{\text{*Var(P}}_{\text{1}}\text{(D))}\\ =\frac{\text{1}}{{\text{P}}_{\text{1}}{\text{(D)}}^{\text{2}}}\text{*}\frac{{\text{P}}_{\text{1}}{\text{(D)*(1-P}}_{\text{1}}\text{(D))}}{{\text{n}}_{\text{1}}}\\ =\frac{{\text{(1-P}}_{\text{1}}\text{(D))}}{{\text{n}}_{\text{1}}{\text{*P}}_{\text{1}}\text{(D)}} \end{array}$$

Substituting the result of equation 4 into equation 3 and we have the final variance for the natural log of the ESR:

(5) $$\begin{array}{l} {\text{Var(ln(ESR}}_{\text{S}}\text{))}\\ =\frac{{\text{(1-P}}_{\text{1}}\text{(D))}}{{\text{n}}_{\text{1}}{\text{*P}}_{\text{1}}\text{(D)}}+\frac{{\text{1-P}}_{\text{2}}\text{(D|E)}}{{\text{P}}_{\text{2}}{\text{(D|E)*n}}_{\text{2E}}}+\frac{{\text{1-P}}_{\text{2}}\text{(D|}\bar{\text{E}}\text{)}}{{\text{P}}_{\text{2}}\text{(D|}\bar{\text{E}}{\text{)*n}}_{\text{2}\bar{\text{E}}}} \end{array}$$

Because we tested our confidence intervals for sample sizes that were 250 or larger, the central limit theorem applies and we can use a normal approximation for estimating the 95% confidence interval.

(6) $$\text{95\% CI}=\text{ exp}\left({\text{ln(ESR}}_{\text{S}}\text{)}\pm \text{1}\text{.96*}\sqrt{{\text{Var(ln(ESR}}_{\text{S}}\text{))}}\right)$$

### Revised Product-Based ESR

Note, from formula 1, we need an estimate of the risk of disease in the unexposed from sample 1 (${\text{P}}_{\text{1}}\text{(D|}\bar{\text{E}})$), rather than the estimate of the overall risk of disease (P_1_(D)). Assuming that the risk of disease in the unexposed is not reported from sample 1 or sample 2, we can use the law of total probability to derive ${\text{P}}_{\text{1}}\text{(D|}\bar{\text{E}})$. By the law of total probability the following formula holds.

(7) $$\begin{array}{l} \text{P(D)}=\text{P(D|E)*P(E)}+\text{P(D|}\bar{\text{E}}\text{)*P(}\bar{\text{E}}\text{)}\\ =\text{RR*P(D|}\bar{\text{E}}\text{)*P(E)}+\text{P(D|}\bar{\text{E}}\text{)*(1-P(E)) }\\ =\text{RR*P(D|}\bar{\text{E}}\text{)*P(E)}+\text{P(D|}\bar{\text{E}}\text{)-(P(D|}\bar{\text{E}}\text{)*P(E))}\\ =\text{(RR-1)*P(D|}\bar{\text{E}}\text{)*P(E)}+\text{P(D|}\bar{\text{E}}\text{)}\\ =\text{P(D|}\bar{\text{E}}\text{)*[((RR-1)*P(E))}+\text{1]} \end{array}$$

Next, solving for $\text{P(D|}\bar{\text{E}}\text{)}$ gives us:

(8) $$\text{P(D|}\bar{\text{E}}\text{)}=\frac{\text{P(D)}}{\text{[((RR-1)*P(E))}+\text{1]}}$$

Here, estimates of P_1_(D)), P_2_(E), and RR_2 _are available in samples one and two as denoted by the subscripts. Then the final estimate for the revised product-based ESR is

(9) $${\text{ESR}}_{\text{R}}=\frac{{\text{P}}_{\text{1}}\text{(D)}}{{\text{[((RR}}_{\text{2}}{\text{-1)*P}}_{\text{2}}\text{(E))}+\text{1]}}*{\text{RR}}_{2}.$$

### Variance and Confidence Interval for the Revised Product-Based ESR

The first estimate of variance derived for the revised product-based is derived for the natural log of the estimate. This is done similar to the derivation for the variance of the natural log of ESR_s _shown in equation 5. The exception is that now we need to find variance of the natural log of the probability of disease among the unexposed in sample 1.

(10) $$\begin{array}{l} {\text{Var(ln(ESR}}_{\text{R}}\text{))}=\\ \frac{{\text{(1-P}}_{\text{1}}\text{(D|}\bar{\text{E}}\text{))}}{{\text{n}}_{\text{1}}{\text{*P}}_{\text{1}}\text{(D|}\bar{\text{E}}\text{)}}+\frac{\text{1}-{\text{P}}_{\text{2}}\text{(D|E)}}{{\text{P}}_{\text{2}}{\text{(D|E)*n}}_{\text{2E}}}+\frac{\text{1}-{\text{P}}_{\text{2}}\text{(D|}\bar{\text{E}}\text{)}}{{\text{P}}_{\text{2}}\text{(D|}\bar{\text{E}}{\text{)*n}}_{\text{2}\bar{\text{E}}}} \end{array}$$

Thus a 95% CI for ESR_R _can be constructed using the normal approximation shown in equation 6 by substituting ESR_R _for ESR_S _and Var(ln(ESR_R_)) for Var(ln(ESR_S_)).

Since the ESR is a probability, the second estimate of the variance derived for ESR_R _is based on the binomial distribution. The variance for a binomial parameter p is $\frac{\text{p*(1}-\text{p)}}{\text{n}}$. We chose to estimate the denominator of this formula by multiplying the sample size from sample 1 by the exposure probability from sample 2. This provides a more conservative estimate of the variance because the denominator will be smaller. The final forms of this variance and the 95% CI using a normal approximation to the binomial distribution are shown below.

(11) $$\text{Var}({\text{ESR}}_{\text{R}})=\frac{{\text{ESR}}_{\text{R}}\text{*(1}-{\text{ESR}}_{\text{R}}\text{)}}{{\text{n}}_{\text{1}}{\text{*P}}_{\text{2}}\text{(E)}}$$

(12) $$\text{95\% CI}={\text{ESR}}_{\text{R}}\pm \text{1}\text{.96*}\sqrt{\frac{{\text{ESR}}_{\text{R}}\text{*(1}-{\text{ESR}}_{\text{R}}\text{)}}{{\text{n}}_{\text{1}}{\text{*P}}_{\text{2}}\text{(E)}}}$$

### Simulation study details

All simulations and subsequent evaluations were performed using SAS statistical software, version 9.2 (SAS, Cary, NC). Populations of size 10 million were generated based on different exposure probabilities, probabilities of disease among the unexposed, and RRs of disease given the exposure. One thousand pairs of samples were drawn from the population to determine the sampling distribution of the overall probability of disease and the RR of disease given exposure. After the samples were generated, estimates of the RR and overall probability of disease (along with their 95% CIs) were calculated for each sample.

For the purposes for this report we organize the results into four scenarios. Scenario 1 considers the situation where the exposure probability was low (.05) and the probability of disease among the unexposed was low (.02). Scenario 2 considers the situation where the exposure probability was low (.05) and the probability of disease among the unexposed was moderate (.09). Scenario 3 considers the situation where the exposure probability was high (.20) and the probability of disease among the unexposed was low (.02). Scenario 4 considers the situation where the exposure probability was high (.20) and the probability of disease among the unexposed was moderate (.09). For all four scenarios we evaluated the properties of the two point estimators and three interval estimators across seven different RRs and four different sample size combinations (Table 1). We chose larger sample sizes because the probability of disease in the unexposed was low (.02) or moderate (.09) and investigators who perform this calculation would want to use the highest quality estimate available. Lastly, in order to evaluate properties of our estimates when the sample size was small, we ran our simulations with a sample size of 250 for both sample 1 and sample 2 under the conditions of Scenario 4 only. We did not perform this analysis in the other three scenarios because the prevalence of exposure and disease among the unexposed was too small to provide a reliable estimate of the RR.

**Table 1:** Parameters varied and all their possible values for the simulation study

Parameter	Possible values
Exposure probability	.05, .20
Probability of disease among unexposed	.02, .09
RR	1.0, 1.5, 2.0, 2.5, 3.0, 4.0, 5.0
Sample size combinations for the overall risk and RR (N_1_/N_2_)	250/250*, 1,000/1,000, 1,000/5,000, 5,000/1,000, 5,000/5,000

*Note: The 250/250 sample size combination was only performed in Scenario 4 (probability of exposure = .20; probability of disease among unexposed = .09).

### Evaluation of ESR Estimators

We calculated the estimated ESR using the simple product-based method and revised product-based method for each of the 1,000 pairs of samples. We evaluated the estimators using observed relative bias. Observed relative bias was defined as the difference between the average of the 1,000 estimates from the 1,000 pairs of samples and the assumed population ESR divided by the assumed population ESR. Observed relative bias can be described as the percent change from the true estimate.

### Evaluation of Confidence Intervals

All 95% CIs were evaluated using coverage probabilities. The coverage probability is defined as the probability that the interval covers the assumed population ESR. For each of the 1,000 pairs of samples we determine whether the assumed population ESR falls between the lower and upper bounds of the CI. The coverage probability is then determined by the number of times the interval covered divided by 1,000. Since we calculated 95% CIs, we expect that our intervals would cover 950 times out of 1,000 (95%).

Expected length given coverage was also evaluated for all of our 95% CIs. For every 95% CI that covered the true value of the ESR for a given pair of 1,000 samples, the length was calculated by subtracting the lower bound from the upper bound. We then calculated the average of these lengths to get the expected length given coverage. For example, if the coverage probability was 95.1% then 951 out of 1,000 intervals covered the true value of the ESR. Therefore the expected length given coverage is based on an *N *of 951. For the purpose of comparison, we also calculated the empirical 95% CI and its length. This was done by examining the distribution of the direct estimator and taking the 2.5^th ^percentile to be the lower bound of the 95% CI and the 97.5^th ^percentile to be the upper bound of the 95% CI. The length of the empirical 95% CI was calculated by subtracting the 2.5^th ^percentile from the 97.5^th ^percentile.

### Case Study

We tested our methodology using a case study in which we calculated the risk of symptomatic knee osteoarthritis (OA) in obese persons by age groups. The overall risk of symptomatic knee OA by age group was derived from Oliveria et al [4]. This article reports on one of the largest population-based studies that estimates the risk of symptomatic knee OA with a cohort of more than 130,000 members of a community health plan. The relative risk of symptomatic knee OA for obese persons (1.91) and proportion obese (0.371) was derived from Niu et al [5]. This study provides one of the most current estimates of the relative risk of symptomatic knee OA by obesity status and also had a substantial sample size (N = 2,660). Since the study by Niu and colleagues only studied those ages 50-79, we limited our analysis to those ages 50-59, 60-69, and 70-79.

## Results

### Scenario 1: Low exposure probability (.05)/Low disease probability among unexposed (.02)

In the case where the probability of exposure was low (.05) and the probability of disease among the unexposed was low (.02), ESR_R _performed better than ESR_S _with respect to observed relative bias. When the RR was 1.0, the observed relative bias was near 0 for both estimators. However, as the RR increased the observed relative bias of ESR_S _increased. This increased to a high of 31.4% when the RR was 5.0 and both sample sizes were 1,000. In the same situation, ESR_R _exhibited an observed relative bias of 3.4%. In general, as the RR increased in magnitude so did the observed relative bias of ESR_S_, while the observed relative bias of ESR_R _was not larger than 4.0% (Table 2).

**Table 2:** Observed relative bias for the simple product-based estimator (ESRS) and the revised product-based estimator (ESRR)

Low exposure probability (.05)/Low disease probability in unexposed (.02)				
	N_1 _= 1,000, N_2 _= 1,000		N_1 _= 5,000, N_2 _= 5,000	
RR/ESR	ESR_S_	ESR_R_	ESR_S_	ESR_R_

1.0/.02	9.5%	3.9%	-1.4%	-2.3%
2.0/.04	7.8%	-2.6%	4.5%	-1.4%
3.0/.06	18.0%	1.8%	12.2%	0.8%
4.0/.08	21.1%	0.0%	17.6%	1.1%
5.0/.10	31.4%	3.4%	22.6%	1.0%

**Low exposure probability (.05)/Moderate disease probability in unexposed (.09)**				
	N_1 _= 1,000, N_2 _= 1,000		N_1 _= 5,000, N_2 _= 5,000	
RR/ESR	ESR_S_	ESR_R_	ESR_S_	ESR_R_

1.0/.09	0.1%	-0.9%	0.9%	0.6%
2.0/.18	6.1%	0.1%	5.5%	0.3%
3.0/.27	9.2%	-1.4%	10.4%	0.2%
4.0/.36	16.6%	0.4%	15.9%	0.5%
5.0/.45	22.0%	0.7%	21.2%	0.8%

**High exposure probability (.20)/Low disease probability in unexposed (.02)**				
	N_1 _= 1,000, N_2 _= 1,000		N_1 _= 5,000, N_2 _= 5,000	
RR/ESR	ESR_S_	ESR_R_	ESR_S_	ESR_R_

1.0/.02	8.7%	1.1%	-0.2%	-1.3%
2.0/.04	26.3%	-1.4%	21.4%	-0.1%
3.0/.06	45.0%	-1.8%	41.7%	0.1%
4.0/.08	73.3%	1.2%	61.6%	0.1%
5.0/.10	93.9%	0.3%	82.5%	0.0%

**High exposure probability (.20)/Moderate disease probability in unexposed (.09)**				
	N_1 _= 1,000, N_2 _= 1,000		N_1 _= 5,000, N_2 _= 5,000	
RR/ESR	ESR_S_	ESR_R_	ESR_S_	ESR_R_

1.0/.09	-0.8%	-0.4%	-1.1%	-1.0%
2.0/.18	22.6%	0.7%	20.4%	0.2%
3.0/.27	40.9%	-0.2%	40.3%	0.0%
4.0/.36	63.6%	0.7%	60.6%	0.1%
5.0/.45	82.6%	0.2%	81.0%	0.2%

N_1 _is the sample the overall risk is derived from

N_2 _is the sample the relative risk is derived from

Relative Risk/Exposure-Specific Risk (RR/ESR) values are the hypothesized values

Coverage probabilities for the 95% CI of ESR_S _were at least 95% when the RR was 2.0 or less, regardless of the sample size combination. However, as the RR increased (and subsequently the observed relative bias), the coverage probabilities began to fall below 95%. The coverage probability fell to 87.1% when the RR was 5.0 and both sample sizes were 5,000 (Table 3). Coverage probabilities for the 95% CI for ESR_R _using a log-based variance were above 95% across all RRs for three of the four sample size combinations (N_1 _= 1,000, N_2 _= 1,000; N_1 _= 5,000, N_2 _= 1,000; and N_1 _= 5,000, N_2 _= 5,000). When the sample size combination was 1,000 for the overall risk (sample 1) and 5,000 for the RR (sample 2) the 95% CI for ESR_R _using a log-based variance failed to attain 95% coverage for all RRs (see additional file 1). The exact opposite relationship was observed for the 95% CI of ESR_R _using a binomial variance. This interval only attained 95% coverage when the sample size combination was 1,000/5,000. In fact, these coverage probabilities well exceeded 95% with the smallest coverage probability being 98.9% when the RR was 1.0 (Table 4).

**Table 3:** Coverage probability for the 95% confidence interval of the simple product-based estimator (ESRS) and revised product-based estimator (ESRR) using a log-based variance

Low exposure probability (.05)/Low disease probability in unexposed (.02)				
	N_1 _= 1,000, N_2 _= 1,000		N_1 _= 5,000, N_2 _= 5,000	
RR/ESR	ESR_S_	ESR_R_	ESR_S_	ESR_R_

1.0/.02	96.8	97.3	97.5	97.5
2.0/.04	96.4	98.1	95.9	97.2
3.0/.06	95.7	98.3	*92.7*	96.6
4.0/.08	*94.1*	98.2	*90.3*	98.0
5.0/.10	*93.9*	98.4	*87.1*	97.7

**Low exposure probability (.05)/Moderate disease probability in unexposed (.09)**				
	N_1 _= 1,000, N_2 _= 1,000		N_1 _= 5,000, N_2 _= 5,000	
RR/ESR	ESR_S_	ESR_R_	ESR_S_	ESR_R_

1.0/.09	96.8	97.2	95.9	96.2
2.0/.18	95.8	96.8	*93.4*	96.3
3.0/.27	*94.3*	97.0	*87.8*	96.4
4.0/.36	*89.3*	96.1	*70.8*	97.0
5.0/.45	*83.2*	96.6	*45.0*	96.6

**High exposure probability (.20)/Low disease probability in unexposed (.02)**				
	N_1 _= 1,000, N_2 _= 1,000		N_1 _= 5,000, N_2 _= 5,000	
RR/ESR	ESR_S_	ESR_R_	ESR_S_	ESR_R_

1.0/.02	97.6	98.5	*94.5*	96.6
2.0/.04	*94.2*	98.9	*85.6*	98.0
3.0/.06	*89.5*	99.0	*55.1*	98.6
4.0/.08	*76.8*	99.2	*22.3*	99.4
5.0/.10	*65.4*	99.6	*4.2*	99.4

**High exposure probability (.20)/Moderate disease probability in unexposed (.09)**				
	N_1 _= 1,000, N_2 _= 1,000		N_1 _= 5,000, N_2 _= 5,000	
RR/ESR	ESR_S_	ESR_R_	ESR_S_	ESR_R_

1.0/.09	*94.7*	96.8	*94.7*	96.5
2.0/.18	*85.4*	98.5	*51.1*	98.0
3.0/.27	*55.9*	98.5	*1.8*	98.6
4.0/.36	*17.6*	99.4	*0*	99.4
5.0/.45	*2.9*	99.5	*0*	99.4

N_1 _is the sample the overall risk is derived from

N_2 _is the sample the relative risk is derived from

Relative Risk/Exposure-Specific Risk (RR/ESR) values are the hypothesized values

Italics denote coverage probabilities that did not attain 95%

**Table 4:** Coverage probability of the 95% confidence interval for the revised product-based estimator (ESRR) using a binomial variance

Low exposure probability (.05)/Low disease probability in unexposed (.02)							
	RR/ESR						
Sample Size (N_1_/N_2_)	1.0/.02	1.5/.03	2.0/.04	2.5/.05	3.0/.06	4.0/.08	5.0/.10

1,000/1,000	*66.1*	*78.1*	*83.2*	*87.1*	*89.6*	*88.9*	*90.0*
1,000/5,000	98.9	99.3	99.6	99.7	99.9	99.5	99.5
5,000/1,000	*54.4*	*60.6*	*61.2*	*62.3*	*63.5*	*64.9*	*65.0*
5,000/5,000	*90.0*	*92.8*	*92.8*	*93.7*	*92.4*	*94.4*	*94.8*

**Low exposure probability (.05)/Moderate disease probability in unexposed (.09)**							
	RR/ESR						
Sample Size (N_1_/N_2_)	1.0/.09	1.5/.14	2.0/.18	2.5/.23	3.0/.27	4.0/.36	5.0/.45

1,000/1,000	*90.4*	*91.6*	*93.2*	*92.4*	*92.2*	*92.0*	*91.5*
1,000/5,000	99.9	100	100	100	99.9	99.7	99.3
5,000/1,000	*62.8*	*63.8*	*63.9*	*63.7*	*62.1*	*61.6*	*62.7*
5,000/5,000	*93.8*	*93.4*	*94.2*	*93.5*	*93.7*	*93.6*	*91.6*

**High exposure probability (.20)/Low disease probability in unexposed (.02)**							
	RR/ESR						
Sample Size (N_1_/N_2_)	1.0/.02	1.5/.03	2.0/.04	2.5/.05	3.0/.06	4.0/.08	5.0/.10

1,000/1,000	*89.1*	*91.2*	*91.8*	*92.6*	*91.5*	*92.5*	*93.8*
1,000/5,000	99.7	99.1	99.4	99.0	98.0	98.8	98.1
5,000/1,000	*63.9*	*68.0*	*64.7*	*69.3*	*70.7*	*70.5*	*71.9*
5,000/5,000	*92.1*	*94.0*	95.1	*94.8*	*94.3*	*94.5*	*93.5*

**High exposure probability (.20)/Moderate disease probability in unexposed (.09)**							
	RR/ESR						
Sample Size (N_1_/N_2_)	1.0/.09	1.5/.14	2.0/.18	2.5/.23	3.0/.27	4.0/.36	5.0/.45

1,000/1,000	*92.5*	*93.2*	95.1	*94.5*	*92.6*	*90.7*	*86.7*
1,000/5,000	99.9	99.7	98.9	98.8	98.2	97.3	95.5
5,000/1,000	*66.0*	*68.3*	*69.7*	*69.0*	*64.4*	*66.6*	*66.0*
5,000/5,000	*94.0*	*94.0*	*93.2*	*94.0*	*91.9*	*92.0*	*88.9*

N_1 _is the sample the overall risk is derived from

N_2 _is the sample the relative risk is derived from

Relative Risk/Exposure-Specific Risk (RR/ESR) values are the hypothesized values

Italics denote coverage probabilities that did not attain 95%

**Figure 1:**
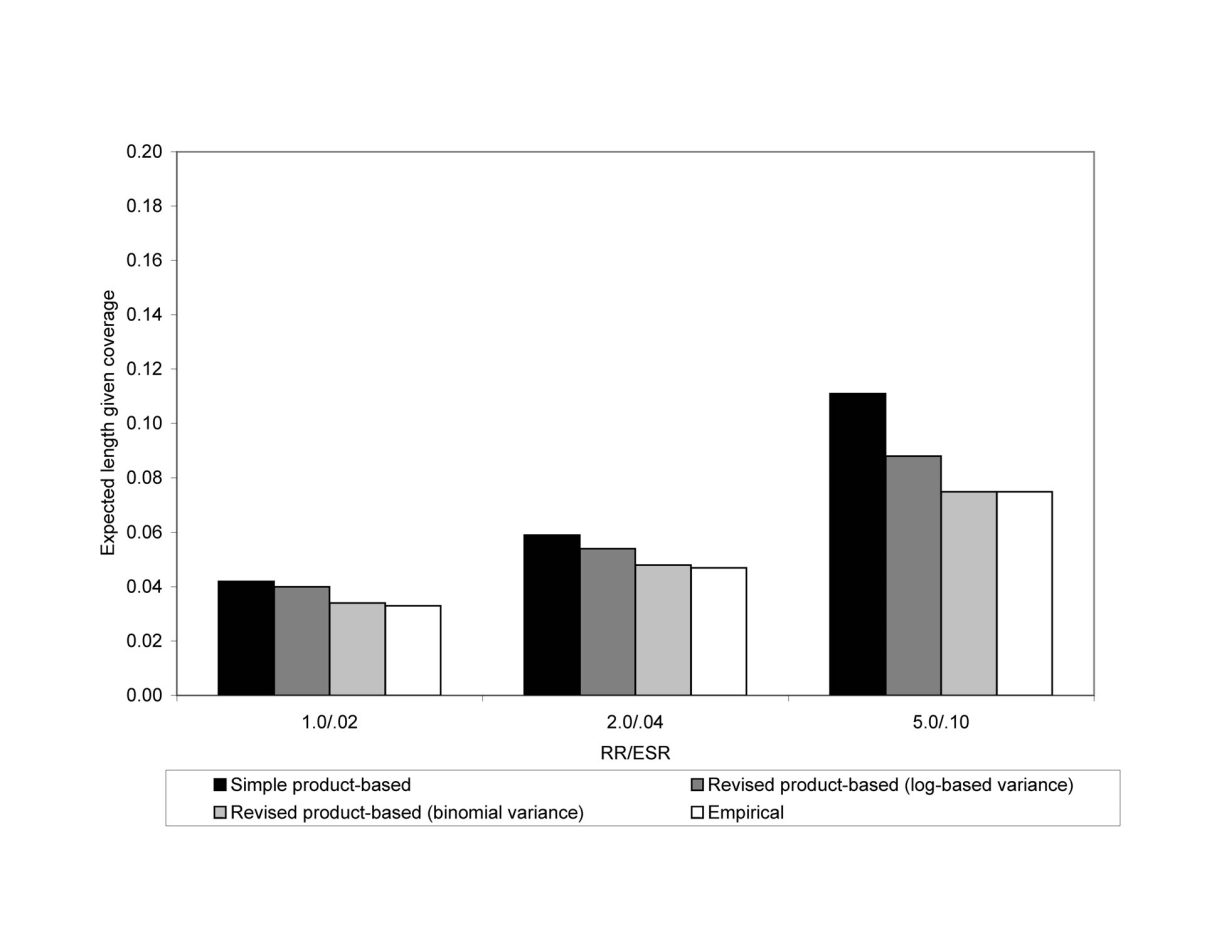
**Expected length given coverage for 95% confidence intervals of the ESR_S_, ESR_R _using a log-based variance, and ESR_R _using a binomial variance in Scenario 1**. Empirical 95% confidence intervals are also shown. The analysis assumed an exposure probability of .05 and risk of disease in the unexposed of .02. The x-axis is the magnitude of the RR. Results are from simulations where both N_1 _and N_2 _are 5,000.

Figure 1 shows the expected lengths given coverage of all the 95% CIs constructed by different RRs (1.0, 2.0, and 5.0 respectively) for the 5,000/5,000 (N_1_/N_2_) sample size combination. The expected length of the empirical 95% CI is also shown. The expected length given coverage is largest for the 95% CI around ESR_S_, and the binomial-based variance yielded 95% CIs around ESR_R _with smaller lengths than the log-based variance 95% CIs in this scenario.

### Scenario 2: Low exposure probability (.05)/Moderate disease probability among unexposed (.09)

Increasing the probability of disease among the unexposed from .02 to .09 while keeping the exposure probability set to .05 did not drastically change our results. The observed relative bias of ESR_S _still increased as the magnitude of the RR increased. When the RR was 5.0, the observed relative bias of ESR_S _was greater than 20% for all sample size combinations. The observed relative bias of ESR_R _was close to zero for all combinations of RR and sample size (Table 2).

Coverage probabilities for the 95% CI of ESR_S _were less than 95% in most cases. The coverage probabilities were adversely affected by the increasing magnitude of the RR with a minimum coverage probability of 45% attained when the RR was 5.0 and the sample size was 5,000 for both samples (Table 3). Similar to Scenario 1, coverage probabilities for the 95% CI of ESR_R _using a log-based variance exhibited at least 95% coverage in all cases except when the sample size the overall risk was derived from was 1,000 and the sample size the RR was derived from was 5,000 (see additional file 1). The 95% CI for ESR_R _using a binomial variance showed the exact opposite relationship. Regardless of the magnitude of the RR, the coverage probability of the 95% CI for ESR_R _using a binomial variance was greater than 99% when the sample size the overall risk was derived from was 1,000 and the sample size the RR was derived from was 5,000 (Table 4).

**Figure 2:**
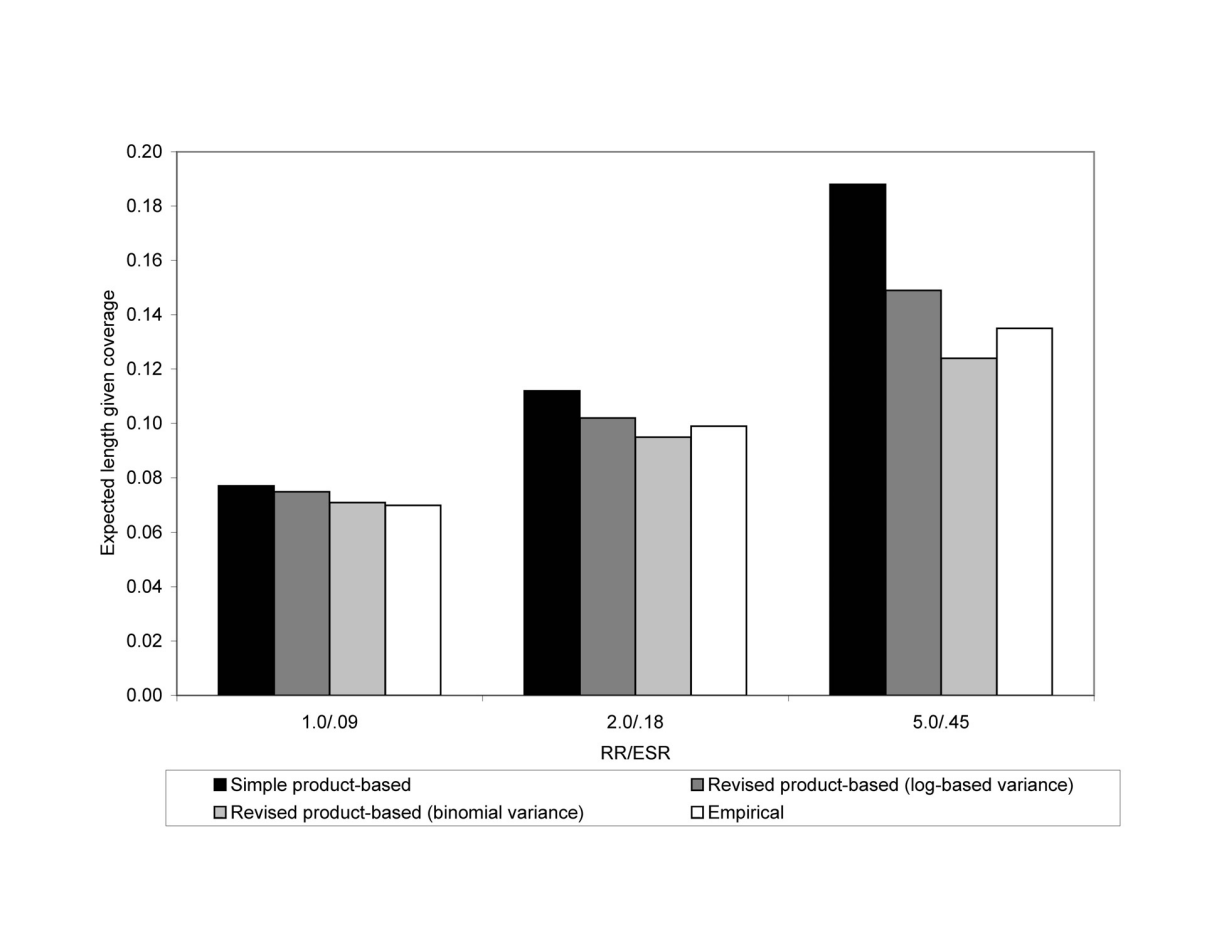
**Expected length given coverage for 95% confidence intervals of the ESR_S_, ESR_R _using a log-based variance, and ESR_R _using a binomial variance in Scenario 2**. Empirical 95% confidence intervals are also shown. The analysis assumed an exposure probability of .05 and risk of disease in the unexposed of .09. The x-axis is the magnitude of the RR. Results are from simulations where both N_1 _and N_2 _are 5,000.

Figure 2 shows the expected lengths given coverage of the 95% CIs. The expected length given coverage increases for all the intervals as the magnitude of the RR increases. The expected lengths of the 95% CIs of ESR_S _and ESR_R _using a log-based variance are similar when the RR is small. However, as the RR increases in magnitude, the length of the 95% CI for ESR_S _is greater than the length of the 95% CI for ESR_R _using a log-based variance.

### Scenario 3: High exposure probability (.20)/Low disease probability among unexposed (.02)

Increasing the exposure probability from .05 to .20 while the probability of disease among the unexposed was .02 affected the results substantially for the existing methodology. The observed relative bias of ESR_S _was over 10% when the RR was 1.5, over 20% when the RR was 2.0, and over 80% when the RR was 5.0. However, the observed relative bias of ESR_R _was near 0% with the greatest observed relative bias being -1.8% when the RR was 3.0 and both sample sizes were 1,000 (Table 2).

In terms of coverage probability, the 95% CI for ESR_S _attained 95% coverage only when the RR was small. When the RR was 5.0, the 95% CI for ESR_S _had a coverage probability as low as 4.2% when the sample size was 5,000 for both samples. Similar to the previous two analyses, coverage probabilities for the 95% CI of ESR_R _using a log-based variance exhibited at least 95% coverage in all cases except when the sample size the overall risk was derived from was 1,000 and the sample size the RR was derived from was 5,000 (see additional file 1). The 95% CI for ESR_R _using a binomial variance showed the exact opposite relationship. Regardless of the magnitude of the RR, the coverage probability of the 95% CI for ESR_R _using a binomial variance was greater than 99% when the sample size the overall risk was derived from was 1,000 and the sample size the RR was derived from was 5,000 (Table 4).

**Figure 3:**
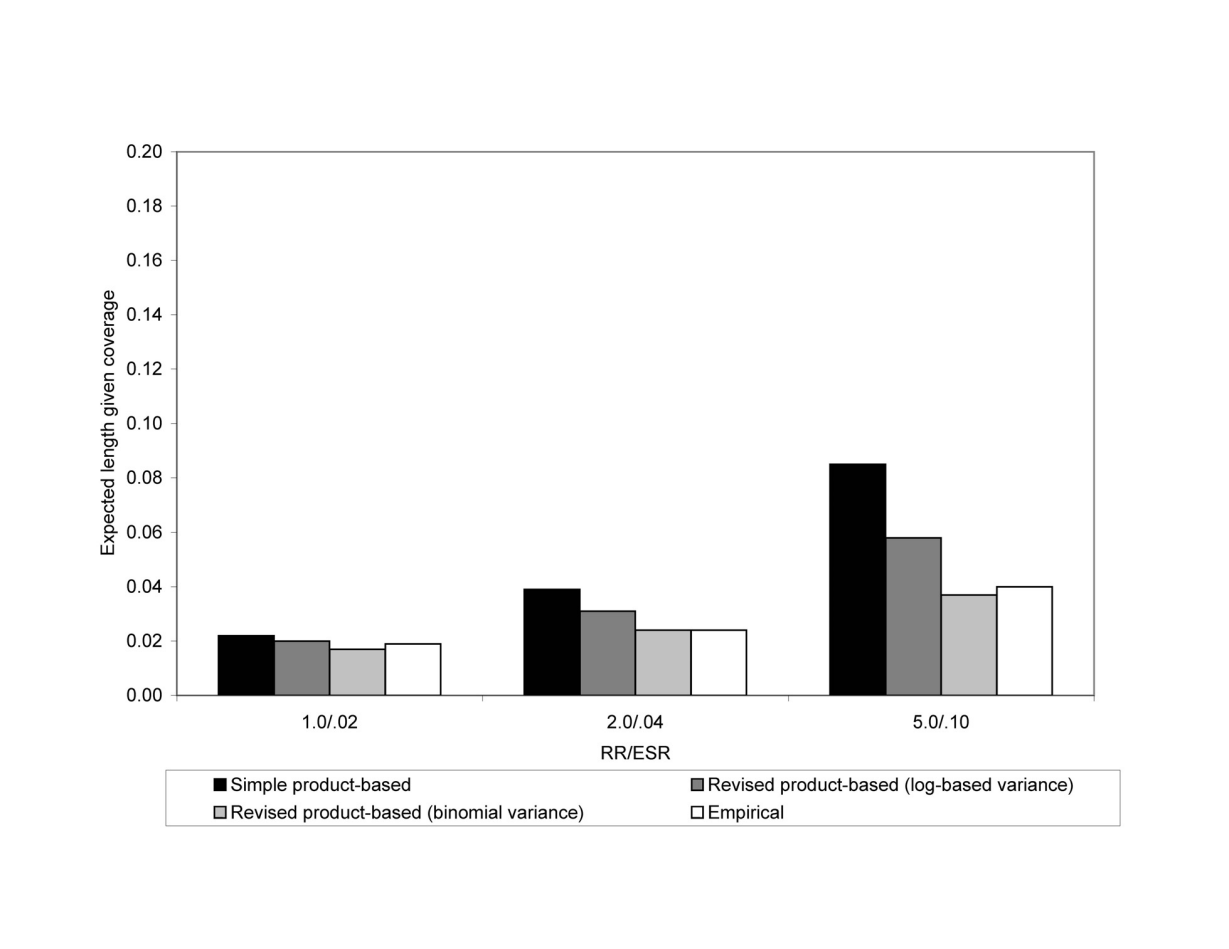
**Expected length given coverage for 95% confidence intervals of the ESR_S_, ESR_R _using a log-based variance, and ESR_R _using a binomial variance in Scenario 3**. Empirical 95% confidence intervals are also shown. The analysis assumed an exposure probability of .20 and risk of disease in the unexposed of .02. The x-axis is the magnitude of the RR. Results are from simulations where both N_1 _and N_2 _are 5,000.

The expected lengths given coverage for Scenario 3 is shown in Figure 3. Compared to Scenario 1 (Figure 1) and Scenario 2 (Figure 2), the expected lengths have decreased substantially. Also, as in Scenario 2, the expected lengths for all of the 95% CIs increased as the magnitude of the RR increased.

### Scenario 4: High exposure probability (.20)/Moderate disease probability among unexposed (.09)

**Figure 4:**
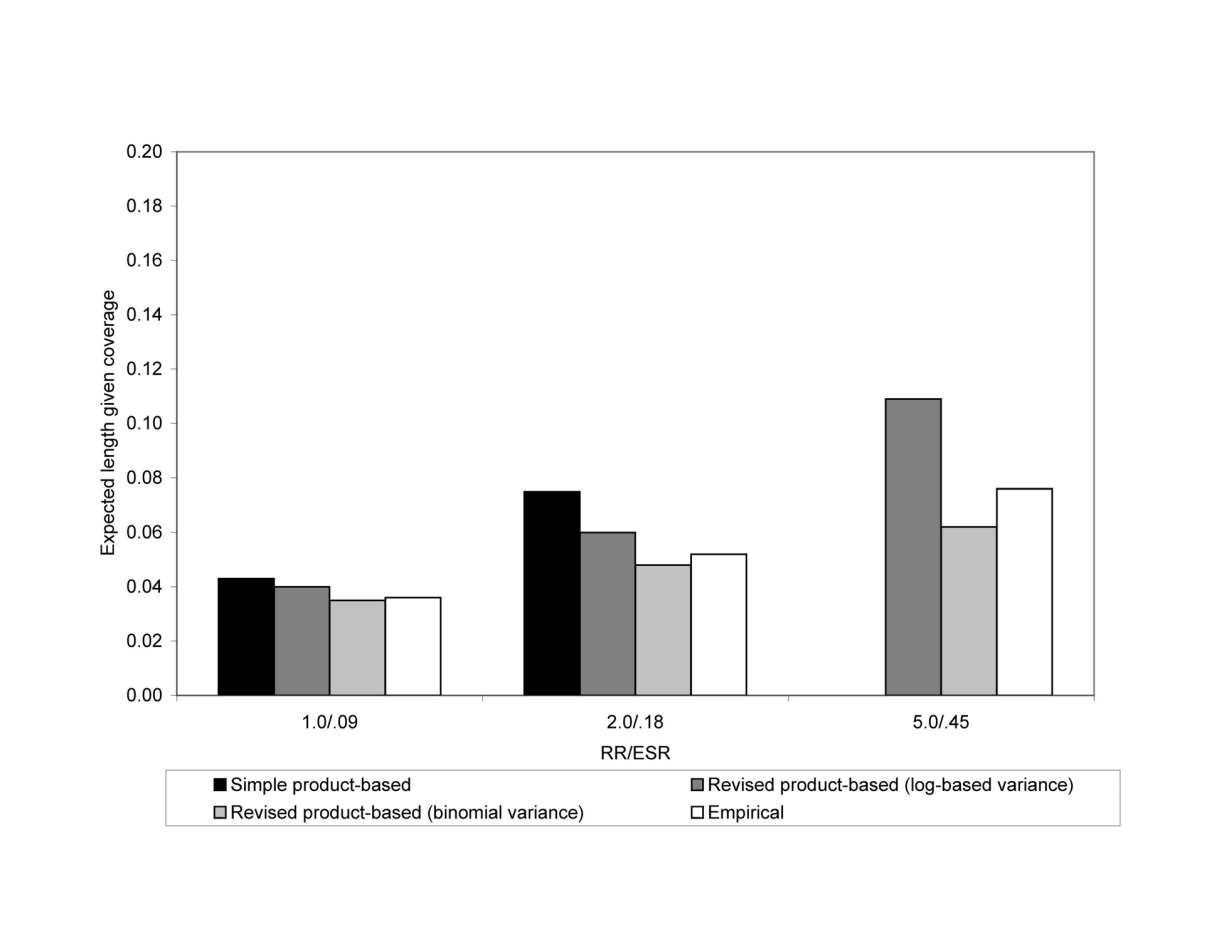
**Expected length given coverage for 95% confidence intervals of the ESR_S_, ESR_R _using a log-based variance, and ESR_R _using a binomial variance in Scenario 4**. Empirical 95% confidence intervals are also shown. The analysis assumed an exposure probability of .20 and risk of disease in the unexposed of .09. The x-axis is the magnitude of the RR. Results are from simulations where both N_1 _and N_2 _are 5,000. Note: The expected length given coverage for the 95% CI around ESR_S _could not be computed when the RR was 5.0 because the coverage probability was 0.

In Scenario 4 we increased both the exposure probability (.20) and the disease probability among the unexposed (.09) at the same time. This gave similar results to Scenario 3. The observed relative bias of ESR_S _increased with increasing RR, while the observed relative bias was near 0% for ESR_R_. Coverage probabilities for the 95% CI of ESR_S _decreased substantially as the RR increased. Coverage probabilities for the 95% CIs for ESR_R _using a log-based variance and binomial variance were not affected by the magnitude of the RR. Expected lengths given coverage also showed similar relationships that were previously described (Figure 4).

In Scenario 4, we also evaluated the properties of our estimator when the sample size was 250 for both samples. We observed similar relationships in terms of observed relative bias and coverage probabilities. The observed relative bias of ESR_S _was 7.8% when the RR was 1.0 and 84.9% when the RR was 5.0, while the observed relative bias of ESR_R _ranged between -1.1% and 1.1%. The coverage probability of the 95% CI for ESR_S _was 96.8% when the RR was 1.0 but fell below 95% when the RR was 1.5 (92.3%) and decreased substantially for a RR of 5.0 (56.8%). The coverage probability of the 95% CI for ESR_R _using a log-based variance was greater than 95% for all RRs. The coverage probability of the 95% CI for ESR_R _using a binomial variance ranged between 85.7 and 92.5%. In terms of expected length given coverage, the 95% CI for ESR_R _using a binomial variance provided shorter intervals and were closer to the length of the empirical interval than the 95% CI using a log-based variance.

### Results of the case study

Results of the case study are shown in Table 5. The estimated risk of symptomatic knee OA was slightly higher when using the simple product-based method. The estimate of the risk of symptomatic knee OA in obese persons ranged between 0.57% and 2.11% when using the revised product-based method. All 95% confidence intervals overlapped with one another for each age group.

**Table 5:** Results from the case study on the risk of symptomatic knee OA in obese persons

Age	Overall risk of symptomatic knee OA in the Oliveria study	Risk of symptomatic knee OA for obese persons using the simple product-based method	95% CI for ESR_S _using a log-based variance	Risk of symptomatic knee OA for obese persons using the revised product-based method	95% CI for ESR_R _using a log-based variance	95% CI for ESR_R _using a binomial variance
50-59	0.0040	0.0076	0.0052-0.0110	0.0057	0.0038-0.0085	0.0037-0.0077
60-69	0.0087	0.0167	0.0121-0.0230	0.0125	0.0089-0.0175	0.0095-0.0155
70-79	0.0147	0.0282	0.0207-0.0383	0.0211	0.0153-0.0289	0.0168-0.0253

*Probability of being obese was derived from the Niu study (0.371)

**RR of symptomatic knee OA for obese persons was derived from the Niu study (1.91)

## Discussion

We have shown via a simulation study that the simple product-based estimator (ESR_S_) that has been calculated in previous studies only performs well in certain situations. Mainly, those situations are when the exposure probability is low (~5%) and the magnitude of the RR is small (~3.0). There are two reasons for this and they can easily be seen by deconstructing the overall risk of disease using the law of total probability.

(13) $$\text{P(D)}=\text{P(D|E)*P(E)}+\text{P(D|}\bar{\text{E}}\text{)*P(}\bar{\text{E}}\text{)}$$

Recall that for the product-based estimator of the ESR to be unbiased that what we really need is an estimate of the risk of disease in the unexposed and not the overall risk. When the exposure probability is low, less weight is put on the probability of disease among the exposed. Put this together with a small RR and most of the overall risk of disease is being influenced by those who are unexposed. However, increasing the exposure probability puts more weight on the risk of disease among the exposed, which will give you a much more biased estimate of the risk of disease among the unexposed. We also showed that ESR_R _provides a substantial improvement over the ESR_S _in terms of observed relative bias. We found that the observed relative bias of ESR_R _was near 0% in almost all cases.

Coverage probabilities for the 95% CI for ESR_S _were inversely related to the observed relative bias of ESR_S_. As the observed relative bias increased, the coverage probability decreased. The overestimation of the ESR using existing methodology (ESR_S_) led to 95% CIs that were less likely to cover the true ESR. Also, the expected lengths given coverage for these 95% CIs were usually longer than the lengths produced for ESR_R _using either the log-based variance or the binomial variance rendering this method of point and interval estimation to be sub-optimal.

Coverage probabilities for the 95% CI for ESR_R _using a log-based variance exhibited greater than 95% coverage in most cases. The exception was when the sample size for the overall risk was 1,000 and the sample size for the RR was 5,000. Paradoxically, this was the only situation in which the 95% CI of ESR_R _using a binomial variance exhibited greater than 95% coverage. In terms of expected length given coverage, neither of these two methods of interval estimation of ESR_R _performed better than the other in all situations. The coverage probability and expected length given coverage depended on the variance estimate that was employed. From equation 11, we can see that the log-based variance of ESR_R _took into account variability from the overall risk and the RR. We also assumed that the two measures were independent and had a covariance of zero, which is a reasonable assumption because the two measures come from two independent samples. From equation 12, we can see that the binomial variance of ESR_R _probability of exposure from sample 2 so that the variance would not be under-estimated. However, in most cases the variability still was under-estimated. When the sample sizes were equal, the under-estimation was very little since the coverage probabilities ranged from 87%-95% in most cases. However in Scenario 1, when the sample size combination was 1,000/1,000 and the RR was 1.0, 1.5, and 2.0 the coverage probabilities were 66%, 78%, and 83% respectively.

The four scenarios, which were defined by the combinations of two different exposure probabilities (.05 and .20) and two different probabilities of disease in the unexposed (.02 and .09), did not affect the observed relative bias of ESR_R_. However, as we increased these two parameters, the observed relative bias of ESR_S _increased. This phenomenon was also demonstrated when comparing coverage probabilities based on the log-based variance for ESR_R _and ESR_S_. When comparing coverage probabilities based on the binomial variance for ESR_R_, the scenario does matter with larger values of the probability of exposure and/or probability of disease in the unexposed increased coverage probabilities. This is not surprising because the estimate of the binomial variance will increase with increasing exposure probabilities and increasing probability of disease among the unexposed.

Results from our case study most closely resemble scenario two where the magnitude of the RR is 2.0. In scenario two, we assumed an exposure probability of 0.20 and a probability of disease in the unexposed of .02. In our case study the RR was 1.91, the exposure probability (probability of being obese) was 0.371, and the overall risk of disease (symptomatic knee OA) ranged from 0.0087 to 0.0132. While the simulations suggest that the estimator would be biased, the overall risk of disease is small so the difference between the two estimates in absolute terms is not large with the largest over-estimation occurring in those ages 70-79 by 0.71%.

It is likely that the estimates produced by Horsburgh and Stewart et al. were accurate. In the article by Horsburgh et al on tuberculosis, he estimated the ESR of tuberculosis for those with advanced HIV infection; old, healed tuberculosis; and immunosuppressive therapy[1]. While the RR of obtaining a new case of tuberculosis is high for those with advanced HIV infection and old, healed tuberculosis, the probability of exposure is so low for these exposures that the impact of the large RR would be muted. For those with immunosuppressive therapy, the RR of a new case of tuberculosis is modest (2.0) and the probability of exposure is low so the overall probability of disease is a good estimate of the probability of disease among those who are not on immunosuppressive therapy [1]. In the Stewart article, the largest RR is 4.62, but this corresponds to an exposure probability of 0.001. When the exposure probabilities are large enough to possibly impact the estimate of the ESR, the RR is low enough (< 2.0) to offset the possible bias [2].

An article by Cupples et al. calculated risk curves for first-degree relatives of patients with Alzheimer's disease. Their method used the odds ratio instead of the relative risk and included converting probabilities to odds [6]. Our method will allow clinicians and other researchers to find the ESR in one step, provided the summary statistics needed for the calculation (P_1_(D), RR_2_, and P_2_(E)) are available.

We acknowledge that there are limitations with this study. The first is that simulation studies can not be considered a proof. However, we did show mathematically that the proposed estimator of the ESR is unbiased and the results of our simulation confirm this finding. It would be important to show mathematically what the true coverage probabilities are for our 95% CIs across different RRs, exposure probabilities, and probabilities of disease among the unexposed. We also acknowledge that our simulations showed coverage probabilities that well exceed 95% when we are calculating 95% CIs for ESR_R _using a log-based variance.

We also evaluated the properties of our point and interval estimators when the sample size was small. We observed that one should only consider carrying out these calculations in smaller samples if the prevalence of exposure and disease among the unexposed is sufficiently large. If one of these values is small than the validity of the estimate of the RR may be questionable. Thus, we recommend that investigators using this methodology only use estimates that are of the highest quality.

The implications of our study are substantial. Clinicians can use these estimates to better explain risk of disease to patients. Many times clinicians and patients can misinterpret the meaning of having a certain RR of disease. Interpreting the probability of disease given a certain exposure (the ESR) is much more transparent. Future studies that examine the calculation of ESRs may look at the impact of having the odds ratio (OR) rather than the RR. Also, the consideration of under which study designs and magnitudes of the exposure/disease would an approximation using the OR be valid is an important question to answer. It is likely that the OR would be valid when the prevalence of the outcome is less than 10% but examining this rigorously would be of great importance [7]. Lastly, re-sampling and bootstrapping techniques may be a useful method of obtaining CIs with appropriate coverage.

## Conclusions

We developed a new estimator for the ESR from two independent samples that exhibits more desirable properties with respect to bias and coverage than the existing methodology. The existing methodology will still perform well when the exposure probability is low. Future methodological studies should focus on the impact of ORs and re-sampling techniques.

## List of abbreviations

ESR: Exposure specific risk; RR: Relative risk; CI: Confidence interval; D: Disease; $\bar{\text{D}}$: Without disease; E: Exposure; $\bar{\text{E}}$: Without exposure; P(): Probability of; Var: Variance; Cov: Covariance; exp(): exponential function; OA: osteoarthritis.

## Competing interests

The authors declare that they have no competing interests.

## Authors' contributions

WMR designed the simulation study, interpreted the data, wrote and critically revised the manuscript, and gave final approval of the manuscript. DG interpreted the data, critically revised the manuscript, and gave final approval of the manuscript. CRH interpreted the data, critically revised the manuscript, and gave final approval of the manuscript. EL designed the simulation study, interpreted the data, critically revised the manuscript, and gave final approval of the manuscript

## Pre-publication history

The pre-publication history for this paper can be accessed here:

http://www.biomedcentral.com/1471-2288/11/1/prepub

## Supplementary Material

Additional file 1 **Tables displaying results for absolute bias, relative bias, and coverage probabilities for all simulation scenarios**. Results from all simulations. Click here for file
