# Supplementary material for: Evaluation of exposure-specific risks from two independent samples: A simulation study
Source: BMC Med Res Methodol. 2011 Jan 5;11:1. doi: 10.1186/1471-2288-11-1 (PMC3022898; doi:10.1186/1471-2288-11-1)
Supplement: Additional file 1 — Tables displaying results for absolute bias, relative bias, and coverage probabilities for all simulation scenarios. Results from all simulations. [file 1471-2288-11-1-S1.DOC]

**Additional files**

Additional file 1

Title: Tables displaying results for absolute bias, relative bias, and coverage probabilities for all simulation scenarios.

Description: Results from all simulations.

Table S1. Observed relative bias for the simple product-based estimator (ESR_S_).

| **Low exposure probability (.05)/Low disease probability in unexposed (.02)** | | | | | | | |
| --- | --- | --- | --- | --- | --- | --- | --- |
|  | RR/ESR | | | | | | |
| Sample Size  (N_1_/N_2_) | 1.0/.02 | 1.5/.03 | 2.0/.04 | 2.5/.05 | 3.0/.06 | 4.0/.08 | 5.0/.10 |
| 1000/1000 | 9.5% | 12.7% | 7.8% | 13.4% | 18.0% | 21.1% | 31.4% |
| 1000/5000 | 1.9% | 1.9% | 5.1% | 10.3% | 14.3% | 15.5% | 19.4% |
| 5000/1000 | 8.3% | 8.7% | 10.2% | 17.3% | 17.3% | 20.2% | 28.9% |
| 5000/5000 | -1.4% | 2.0% | 4.5% | 11.2% | 12.2% | 17.6% | 22.6% |
| **Low exposure probability (.05)/Moderate disease probability in unexposed (.09)** | | | | | | | |
|  | RR/ESR | | | | | | |
| Sample Size  (N_1_/N_2_) | 1.0/.09 | 1.5/.14 | 2.0/.18 | 2.5/.23 | 3.0/.27 | 4.0/.36 | 5.0/.45 |
| 1000/1000 | 0.1% | 2.7% | 6.1% | 8.7% | 9.2% | 16.6% | 22.0% |
| 1000/5000 | 2.0% | 1.6% | 5.9% | 7.7% | 10.3% | 14.9% | 20.0% |
| 5000/1000 | 0.5% | 7.0% | 7.0% | 8.3% | 10.8% | 17.1% | 20.1% |
| 5000/5000 | 0.9% | 3.5% | 5.5% | 7.8% | 10.4% | 15.9% | 21.2% |
| **High exposure probability (.20)/Low disease probability in unexposed (.02)** | | | | | | | |
|  | RR/ESR | | | | | | |
| Sample Size  (N_1_/N_2_) | 1.0/.02 | 1.5/.03 | 2.0/.04 | 2.5/.05 | 3.0/.06 | 4.0/.08 | 5.0/.10 |
| 1000/1000 | 8.7% | 20.4% | 26.3% | 39.2% | 45.0% | 73.3% | 93.9% |
| 1000/5000 | 1.7% | 11.3% | 22.7% | 31.7% | 42.4% | 62.0% | 82.0% |
| 5000/1000 | 8.2% | 18.2% | 31.2% | 39.6% | 46.4% | 73.1% | 92.8% |
| 5000/5000 | -0.2% | 12.0% | 21.4% | 28.9% | 41.7% | 61.6% | 82.5% |
| **High exposure probability (.20)/Moderate disease probability in unexposed (.09)** | | | | | | | |
|  | RR/ESR | | | | | | |
| Sample Size  (N_1_/N_2_) | 1.0/.09 | 1.5/.14 | 2.0/.18 | 2.5/.23 | 3.0/.27 | 4.0/.36 | 5.0/.45 |
| 1000/1000 | 0.8% | 11.8% | 22.6% | 31.9% | 40.9% | 63.6% | 82.6% |
| 1000/5000 | 0.6% | 10.8% | 21.0% | 29.7% | 40.4% | 59.1% | 81.5% |
| 5000/1000 | -0.4% | 12.3% | 23.0% | 32.8% | 40.5% | 61.0% | 83.6% |
| 5000/5000 | -1.1% | 10.3% | 20.4% | 30.2% | 40.3% | 60.6% | 81.0% |

N_1_ is the sample the overall risk is derived from

N_2_ is the sample the relative risk is derived from

Relative Risk/Exposure-Specific Risk (RR/ESR) values are the hypothesized values

Relative Risk/Exposure-Specific Risk values are the hypothesized values

Table S2. Observed relative bias for the revised product-based estimator (ESR_R_).

| **Low exposure probability (.05)/Low disease probability in unexposed (.02)** | | | | | | | |
| --- | --- | --- | --- | --- | --- | --- | --- |
|  | RR/ESR | | | | | | |
| Sample Size  (N_1_/N_2_) | 1.0/.02 | 1.5/.03 | 2.0/.04 | 2.5/.05 | 3.0/.06 | 4.0/.08 | 5.0/.10 |
| 1000/1000 | 3.9% | 4.0% | -2.6% | 0.1% | 1.8% | 0.0% | 3.4% |
| 1000/5000 | 0.8% | -1.6% | -0.9% | 1.5% | 2.7% | -0.3 | -1.4% |
| 5000/1000 | 2.9% | 0.4% | -0.1% | 3.0% | 1.2% | -0.6% | 1.8% |
| 5000/5000 | -2.3% | -1.4% | -1.4% | 2.2% | 0.8% | 1.1% | 1.0% |
| **Low exposure probability (.05)/Moderate disease probability in unexposed (.09)** | | | | | | | |
|  | RR/ESR | | | | | | |
| Sample Size  (N_1_/N_2_) | 1.0/.09 | 1.5/.14 | 2.0/.18 | 2.5/.23 | 3.0/.27 | 4.0/.36 | 5.0/.45 |
| 1000/1000 | -0.9% | -0.9% | 0.1% | 0.2% | -1.4% | 0.4% | 0.7% |
| 1000/5000 | 1.7% | -1.0% | 0.6% | 0.0% | 0.1% | -0.3% | -0.2% |
| 5000/1000 | -0.5% | 3.1% | 0.9% | -0.2% | -0.2% | 0.8% | -0.3% |
| 5000/5000 | 0.6% | 0.8% | 0.3% | 0.0% | 0.2% | 0.5% | 0.8% |
| **High exposure probability (.20)/Low disease probability in unexposed (.02)** | | | | | | | |
|  | RR/ESR | | | | | | |
| Sample Size  (N_1_/N_2_) | 1.0/.02 | 1.5/.03 | 2.0/.04 | 2.5/.05 | 3.0/.06 | 4.0/.08 | 5.0/.10 |
| 1000/1000 | 1.1% | 1.7% | -1.4% | 0.4% | -1.8% | 1.2% | 0.3% |
| 1000/5000 | 0.4% | -0.4% | 0.8% | 0.3% | 0.3% | -0.1% | -0.4% |
| 5000/1000 | 0.7% | 0.7% | 1.2% | 0.2% | -1.0% | 0.9% | 0.2% |
| 5000/5000 | -1.3% | 0.4% | -0.1% | -1.4% | 0.1% | 0.1% | 0.0% |
| **High exposure probability (.20)/Moderate disease probability in unexposed (.09)** | | | | | | | |
|  | Relative Risk/Probability of disease in exposed | | | | | | |
| Sample Size  (N_1_/N_2_) | 1.0/.09 | 1.5/.14 | 2.0/.18 | 2.5/.23 | 3.0/.27 | 4.0/.36 | 5.0/.45 |
| 1000/1000 | -0.4% | 0.3% | 0.7% | 0.2% | -0.2% | 0.7% | 0.2% |
| 1000/5000 | 0.3% | 0.4% | 0.6% | -0.4% | 0.2% | -0.6% | 0.4% |
| 5000/1000 | -1.3% | 0.7% | 0.9% | 0.5% | -0.4% | -0.2% | 0.6% |
| 5000/5000 | -1.0% | 0.0% | 0.2% | 0.0% | 0.0% | 0.1% | 0.2% |

N_1_ is the sample the overall risk is derived from

N_2_ is the sample the relative risk is derived from

Relative Risk/Exposure-Specific Risk (RR/ESR) values are the hypothesized values

Relative Risk/Exposure-Specific Risk values are the hypothesized values

Table S3. Observed absolute bias for the simple product-based estimator (ESR_S_).

| **Low exposure probability (.05)/Low disease probability in unexposed (.02)** | | | | | | | |
| --- | --- | --- | --- | --- | --- | --- | --- |
|  | RR/ESR | | | | | | |
| Sample Size  (N_1_/N_2_) | 1.0/.02 | 1.5/.03 | 2.0/.04 | 2.5/.05 | 3.0/.06 | 4.0/.08 | 5.0/.10 |
| 1000/1000 | 0.002 | 0.004 | 0.003 | 0.007 | 0.011 | 0.017 | 0.031 |
| 1000/5000 | 0.000 | 0.001 | 0.002 | 0.005 | 0.009 | 0.012 | 0.019 |
| 5000/1000 | 0.002 | 0.003 | 0.004 | 0.009 | 0.010 | 0.016 | 0.029 |
| 5000/5000 | 0.000 | 0.001 | 0.002 | 0.006 | 0.007 | 0.014 | 0.023 |
| **Low exposure probability (.05)/Moderate disease probability in unexposed (.09)** | | | | | | | |
|  | RR/ESR | | | | | | |
| Sample Size  (N_1_/N_2_) | 1.0/.09 | 1.5/.14 | 2.0/.18 | 2.5/.23 | 3.0/.27 | 4.0/.36 | 5.0/.45 |
| 1000/1000 | 0.000 | 0.004 | 0.011 | 0.020 | 0.025 | 0.060 | 0.099 |
| 1000/5000 | 0.002 | 0.002 | 0.011 | 0.017 | 0.028 | 0.054 | 0.090 |
| 5000/1000 | 0.000 | 0.009 | 0.013 | 0.019 | 0.029 | 0.061 | 0.090 |
| 5000/5000 | 0.001 | 0.005 | 0.010 | 0.017 | 0.028 | 0.057 | 0.096 |
| **High exposure probability (.20)/Low disease probability in unexposed (.02)** | | | | | | | |
|  | RR/ESR | | | | | | |
| Sample Size  (N_1_/N_2_) | 1.0/.02 | 1.5/.03 | 2.0/.04 | 2.5/.05 | 3.0/.06 | 4.0/.08 | 5.0/.10 |
| 1000/1000 | 0.002 | 0.006 | 0.011 | 0.020 | 0.027 | 0.059 | 0.094 |
| 1000/5000 | 0.000 | 0.003 | 0.009 | 0.016 | 0.025 | 0.050 | 0.082 |
| 5000/1000 | 0.002 | 0.005 | 0.012 | 0.020 | 0.028 | 0.058 | 0.093 |
| 5000/5000 | 0.000 | 0.004 | 0.009 | 0.014 | 0.025 | 0.049 | 0.082 |
| **High exposure probability (.20)/Moderate disease probability in unexposed (.09)** | | | | | | | |
|  | RR/ESR | | | | | | |
| Sample Size  (N_1_/N_2_) | 1.0/.09 | 1.5/.14 | 2.0/.18 | 2.5/.23 | 3.0/.27 | 4.0/.36 | 5.0/.45 |
| 1000/1000 | 0.001 | 0.016 | 0.041 | 0.072 | 0.111 | 0.229 | 0.372 |
| 1000/5000 | 0.001 | 0.015 | 0.038 | 0.067 | 0.109 | 0.213 | 0.367 |
| 5000/1000 | 0.000 | 0.017 | 0.041 | 0.074 | 0.109 | 0.220 | 0.376 |
| 5000/5000 | -0.001 | 0.014 | 0.037 | 0.068 | 0.109 | 0.218 | 0.364 |

N_1_ is the sample the overall risk is derived from

N_2_ is the sample the relative risk is derived from

Relative Risk/Exposure-Specific Risk (RR/ESR) values are the hypothesized values

Relative Risk/Exposure-Specific Risk values are the hypothesized values

Table S4. Observed absolute bias for the revised product-based estimator (ESR_R_).

| **Low exposure probability (.05)/Low disease probability in unexposed (.02)** | | | | | | | |
| --- | --- | --- | --- | --- | --- | --- | --- |
|  | RR/ESR | | | | | | |
| Sample Size  (N_1_/N_2_) | 1.0/.02 | 1.5/.03 | 2.0/.04 | 2.5/.05 | 3.0/.06 | 4.0/.08 | 5.0/.10 |
| 1000/1000 | 0.001 | 0.001 | -0.001 | 0.000 | 0.001 | 0.000 | 0.003 |
| 1000/5000 | 0.000 | 0.000 | 0.000 | 0.001 | 0.002 | 0.000 | -0.001 |
| 5000/1000 | 0.001 | 0.000 | 0.000 | 0.002 | 0.001 | 0.000 | 0.002 |
| 5000/5000 | 0.000 | 0.000 | -0.001 | 0.001 | 0.001 | 0.001 | 0.001 |
| **Low exposure probability (.05)/Moderate disease probability in unexposed (.09)** | | | | | | | |
|  | RR/ESR | | | | | | |
| Sample Size  (N_1_/N_2_) | 1.0/.09 | 1.5/.14 | 2.0/.18 | 2.5/.23 | 3.0/.27 | 4.0/.36 | 5.0/.45 |
| 1000/1000 | -0.001 | -0.001 | 0.000 | 0.000 | -0.004 | 0.001 | 0.003 |
| 1000/5000 | 0.002 | -0.001 | 0.001 | 0.000 | 0.000 | -0.001 | -0.001 |
| 5000/1000 | 0.000 | 0.004 | 0.002 | -0.001 | -0.001 | 0.003 | -0.001 |
| 5000/5000 | 0.001 | 0.001 | 0.001 | 0.000 | 0.000 | 0.002 | 0.004 |
| **High exposure probability (.20)/Low disease probability in unexposed (.02)** | | | | | | | |
|  | RR/ESR | | | | | | |
| Sample Size  (N_1_/N_2_) | 1.0/.02 | 1.5/.03 | 2.0/.04 | 2.5/.05 | 3.0/.06 | 4.0/.08 | 5.0/.10 |
| 1000/1000 | 0.000 | 0.000 | -0.001 | 0.000 | -0.001 | 0.001 | 0.000 |
| 1000/5000 | 0.000 | 0.000 | 0.000 | 0.000 | 0.000 | 0.000 | 0.000 |
| 5000/1000 | 0.000 | 0.000 | 0.000 | 0.000 | -0.001 | 0.001 | 0.000 |
| 5000/5000 | 0.000 | 0.000 | 0.000 | -0.001 | 0.000 | 0.000 | 0.000 |
| **High exposure probability (.20)/Moderate disease probability in unexposed (.09)** | | | | | | | |
|  | RR/ESR | | | | | | |
| Sample Size  (N_1_/N_2_) | 1.0/.09 | 1.5/.14 | 2.0/.18 | 2.5/.23 | 3.0/.27 | 4.0/.36 | 5.0/.45 |
| 1000/1000 | 0.000 | 0.000 | 0.001 | 0.000 | -0.001 | 0.003 | 0.001 |
| 1000/5000 | 0.000 | 0.001 | 0.001 | -0.001 | 0.000 | -0.002 | 0.002 |
| 5000/1000 | -0.001 | 0.001 | 0.002 | 0.001 | -0.001 | -0.001 | 0.003 |
| 5000/5000 | -0.001 | 0.000 | 0.000 | 0.000 | 0.000 | 0.000 | 0.001 |

N_1_ is the sample the overall risk is derived from

N_2_ is the sample the relative risk is derived from

Relative Risk/Exposure-Specific Risk (RR/ESR) values are the hypothesized values

Relative Risk/Exposure-Specific Risk values are the hypothesized values

Table S5. Coverage probability for the 95% confidence interval of the simple product-based estimator (ESR_S_).

| **Low exposure probability (.05)/Low disease probability in unexposed (.02)** | | | | | | | |
| --- | --- | --- | --- | --- | --- | --- | --- |
|  | RR/ESR | | | | | | |
| Sample Size  (N_1_/N_2_) | 1.0/.02 | 1.5/.03 | 2.0/.04 | 2.5/.05 | 3.0/.06 | 4.0/.08 | 5.0/.10 |
| 1000/1000 | 96.8 | 96.1 | 96.4 | 96.4 | 95.7 | *94.1* | *93.9* |
| 1000/5000 | 96.5 | 96.1 | 96.0 | 95.0 | *94.1* | *92.1* | *91.9* |
| 5000/1000 | 96.6 | 95.4 | 96.5 | *94.9* | 95.1 | *93.8* | *93.3* |
| 5000/5000 | 97.5 | 97.4 | 95.9 | *93.7* | *92.7* | *90.3* | *87.1* |
| **Low exposure probability (.05)/Moderate disease probability in unexposed (.09)** | | | | | | | |
|  | RR/ESR | | | | | | |
| Sample Size  (N_1_/N_2_) | 1.0/.09 | 1.5/.14 | 2.0/.18 | 2.5/.23 | 3.0/.27 | 4.0/.36 | 5.0/.45 |
| 1000/1000 | 96.8 | 95.0 | 95.8 | *93.0* | *94.3* | *89.3* | *83.2* |
| 1000/5000 | *94.8* | 95.1 | *94.0* | *91.4* | *89.7* | *82.9* | *69.8* |
| 5000/1000 | 96.7 | 95.2 | 95.0 | *92.7* | *91.7* | *86.1* | *85.0* |
| 5000/5000 | 95.9 | *94.1* | *93.4* | *90.4* | *87.8* | *70.8* | *45.0* |
| **High exposure probability (.20)/Low disease probability in unexposed (.02)** | | | | | | | |
|  | RR/ESR | | | | | | |
| Sample Size  (N_1_/N_2_) | 1.0/.02 | 1.5/.03 | 2.0/.04 | 2.5/.05 | 3.0/.06 | 4.0/.08 | 5.0/.10 |
| 1000/1000 | 97.6 | *94.7* | *94.2* | *91.7* | *89.5* | *76.8* | *65.4* |
| 1000/5000 | 95.7 | *94.0* | *88.6* | *82.2* | *71.8* | *50.4* | *26.2* |
| 5000/1000 | 96.9 | 96.2 | *92.2* | *90.5* | *87.4* | *72.9* | *58.9* |
| 5000/5000 | *94.5* | *93.2* | *85.6* | *77.7* | *55.1* | *22.3* | *4.2* |
| **High exposure probability (.20)/Moderate disease probability in unexposed (.09)** | | | | | | | |
|  | RR/ESR | | | | | | |
| Sample Size  (N_1_/N_2_) | 1.0/.09 | 1.5/.14 | 2.0/.18 | 2.5/.23 | 3.0/.27 | 4.0/.36 | 5.0/.45 |
| 1000/1000 | *94.7* | *92.4* | *85.4* | *73.3* | *55.9* | *17.6* | *2.9* |
| 1000/5000 | *94.3* | *89.3* | *67.9* | *42.4* | *14.5* | *0.7* | *0* |
| 5000/1000 | 96.1 | *92.6* | *83.1* | *65.6* | *50.9* | *14.3* | *1.1* |
| 5000/5000 | *94.7* | 84.0 | *51.1* | *16.0* | *1.8* | *0* | *0* |

N_1_ is the sample the overall risk is derived from

N_2_ is the sample the relative risk is derived from

Relative Risk/Exposure-Specific Risk (RR/ESR) values are the hypothesized values

Italics denote coverage probabilities that did not attain 95%

Table S6. Coverage probability for the 95% confidence interval of the revised product-based estimator (ESR_R_) using a log-based variance.

| **Low exposure probability (.05)/Low disease probability in unexposed (.02)** | | | | | | | |
| --- | --- | --- | --- | --- | --- | --- | --- |
|  | RR/ESR | | | | | | |
| Sample Size  (N_1_/N_2_) | 1.0/.02 | 1.5/.03 | 2.0/.04 | 2.5/.05 | 3.0/.06 | 4.0/.08 | 5.0/.10 |
| 1000/1000 | 97.3 | 96.9 | 98.1 | 98.1 | 98.3 | 98.2 | 98.4 |
| 1000/5000 | *94.7* | *93.5* | *92.8* | *92.8* | *91.0* | *89.0* | *87.6* |
| 5000/1000 | 97.5 | 97.9 | 98.3 | 98.1 | 98.2 | 99.4 | 99.6 |
| 5000/5000 | 97.5 | 98.0 | 97.2 | 96.7 | 96.6 | 98.0 | 97.7 |
| **Low exposure probability (.05)/Moderate disease probability in unexposed (.09)** | | | | | | | |
|  | RR/ESR | | | | | | |
| Sample Size  (N_1_/N_2_) | 1.0/.09 | 1.5/.14 | 2.0/.18 | 2.5/.23 | 3.0/.27 | 4.0/.36 | 5.0/.45 |
| 1000/1000 | 97.2 | 96.5 | 96.8 | 97.0 | 97.0 | 96.9 | 96.6 |
| 1000/5000 | *93.4* | *92.8* | *90.8* | *89.5* | *89.8* | *88.4* | *87.1* |
| 5000/1000 | 97.9 | 97.4 | 97.5 | 96.6 | 97.2 | 98.2 | 97.8 |
| 5000/5000 | 96.2 | 95.5 | 96.3 | 96.2 | 96.4 | 97.0 | 96.6 |
| **High exposure probability (.20)/Low disease probability in unexposed (.02)** | | | | | | | |
|  | RR/ESR | | | | | | |
| Sample Size  (N_1_/N_2_) | 1.0/.02 | 1.5/.03 | 2.0/.04 | 2.5/.05 | 3.0/.06 | 4.0/.08 | 5.0/.10 |
| 1000/1000 | 98.5 | 98.9 | 98.9 | 98.6 | 99.0 | 99.2 | 99.6 |
| 1000/5000 | 90.5 | 87.5 | 87.3 | 86.7 | 87.2 | 89.4 | 90.4 |
| 5000/1000 | 99.1 | 99.9 | 100 | 99.7 | 100 | 99.9 | 100 |
| 5000/5000 | 96.6 | 98.1 | 98.0 | 98.8 | 98.6 | 99.4 | 99.4 |
| **High exposure probability (.20)/Moderate disease probability in unexposed (.09)** | | | | | | | |
|  | RR/ESR | | | | | | |
| Sample Size  (N_1_/N_2_) | 1.0/.09 | 1.5/.14 | 2.0/.18 | 2.5/.23 | 3.0/.27 | 4.0/.36 | 5.0/.45 |
| 1000/1000 | 96.8 | 97.3 | 98.5 | 99.2 | 98.5 | 99.4 | 99.5 |
| 1000/5000 | *89.4* | *89.3* | *88.8* | *88.9* | *89.2* | *85.6* | *87.7* |
| 5000/1000 | 99.2 | 99.2 | 99.6 | 99.9 | 99.8 | 99.6 | 100 |
| 5000/5000 | 96.5 | 97.9 | 98.0 | 98.5 | 98.6 | 99.4 | 99.4 |

N_1_ is the sample the overall risk is derived from

N_2_ is the sample the relative risk is derived from

Relative Risk/Exposure-Specific Risk (RR/ESR) values are the hypothesized values

Italics denote coverage probabilities that did not attain 95%

Table S7. Coverage probability of the 95% confidence interval for the revised product-based estimator (ESR_R_) using a binomial variance.

| **Low exposure probability (.05)/Low disease probability in unexposed (.02)** | | | | | | | |
| --- | --- | --- | --- | --- | --- | --- | --- |
|  | RR/ESR | | | | | | |
| Sample Size  (N_1_/N_2_) | 1.0/.02 | 1.5/.03 | 2.0/.04 | 2.5/.05 | 3.0/.06 | 4.0/.08 | 5.0/.10 |
| 1000/1000 | *66.1* | *78.1* | *83.2* | *87.1* | *89.6* | *88.9* | *90.0* |
| 1000/5000 | 98.9 | 99.3 | 99.6 | 99.7 | 99.9 | 99.5 | 99.5 |
| 5000/1000 | *54.4* | *60.6* | *61.2* | *62.3* | *63.5* | *64.9* | *65.0* |
| 5000/5000 | *90.0* | *92.8* | *92.8* | *93.7* | *92.4* | *94.4* | *94.8* |
| **Low exposure probability (.05)/Moderate disease probability in unexposed (.09)** | | | | | | | |
|  | RR/ESR | | | | | | |
| Sample Size  (N_1_/N_2_) | 1.0/.09 | 1.5/.14 | 2.0/.18 | 2.5/.23 | 3.0/.27 | 4.0/.36 | 5.0/.45 |
| 1000/1000 | *90.4* | *91.6* | *93.2* | *92.4* | *92.2* | *92.0* | *91.5* |
| 1000/5000 | 99.9 | 100 | 100 | 100 | 99.9 | 99.7 | 99.3 |
| 5000/1000 | *62.8* | *63.8* | *63.9* | *63.7* | *62.1* | *61.6* | *62.7* |
| 5000/5000 | *93.8* | *93.4* | *94.2* | *93.5* | *93.7* | *93.6* | *91.6* |
| **High exposure probability (.20)/Low disease probability in unexposed (.02)** | | | | | | | |
|  | RR/ESR | | | | | | |
| Sample Size  (N_1_/N_2_) | 1.0/.02 | 1.5/.03 | 2.0/.04 | 2.5/.05 | 3.0/.06 | 4.0/.08 | 5.0/.10 |
| 1000/1000 | *89.1* | *91.2* | *91.8* | *92.6* | *91.5* | *92.5* | *93.8* |
| 1000/5000 | 99.7 | 99.1 | 99.4 | 99.0 | 98.0 | 98.8 | 98.1 |
| 5000/1000 | *63.9* | *68.0* | *64.7* | *69.3* | *70.7* | *70.5* | *71.9* |
| 5000/5000 | *92.1* | *94.0* | 95.1 | *94.8* | *94.3* | *94.5* | *93.5* |
| **High exposure probability (.20)/Moderate disease probability in unexposed (.09)** | | | | | | | |
|  | RR/ESR | | | | | | |
| Sample Size  (N_1_/N_2_) | 1.0/.09 | 1.5/.14 | 2.0/.18 | 2.5/.23 | 3.0/.27 | 4.0/.36 | 5.0/.45 |
| 1000/1000 | *92.5* | *93.2* | 95.1 | *94.5* | *92.6* | *90.7* | *86.7* |
| 1000/5000 | 99.9 | 99.7 | 98.9 | 98.8 | 98.2 | 97.3 | 95.5 |
| 5000/1000 | *66.0* | *68.3* | *69.7* | *69.0* | *64.4* | *66.6* | *66.0* |
| 5000/5000 | *94.0* | *94.0* | *93.2* | *94.0* | *91.9* | *92.0* | *88.9* |

N_1_ is the sample the overall risk is derived from

N_2_ is the sample the relative risk is derived from

Relative Risk/Exposure-Specific Risk (RR/ESR) values are the hypothesized values

Italics denote coverage probabilities that did not attain 95%
